# Supplementary material for: Oxygen Vacancy and Interface Effect Adjusted Hollow Dodecahedrons for Efficient Oxygen Evolution Reaction
Source: Molecules. 2023 Jul 25;28(15):5620. doi: 10.3390/molecules28155620 (PMC10419998; doi:10.3390/molecules28155620)
Supplement: Supplementary file 1 [file molecules-28-05620-s001.zip › molecules-2499211-supplementary.pdf]

Supporting information

Oxygen vacancy and interface effect adjusted hollow dodecahedrons for efficient oxygen evolution reaction

Huan Wang[1], Qian Ma[1], Fengmin Sun[1], Yachuan Shao[1], Di Zhang[1], Huilan Sun[1], Zhaojin Li[1], Qiujun Wang[1], Jian Qi[2,*], Bo Wang[1,*]

[1] *Hebei Key Laboratory of Flexible Functionals Materials, School of Materials Science and Engineering, Hebei University of Science and Technology, Shijiazhuang 050000, PR China.*

[2] *State Key Laboratory of Biochemical Engineering, Institute of Process Engineering, Chinese Academy of Sciences, Beijing 100049, PR China.*

* *Correspondence: jqi@ipe.ac.cn (J.Q.), wangbo1996@gmail.com (B.W.)*

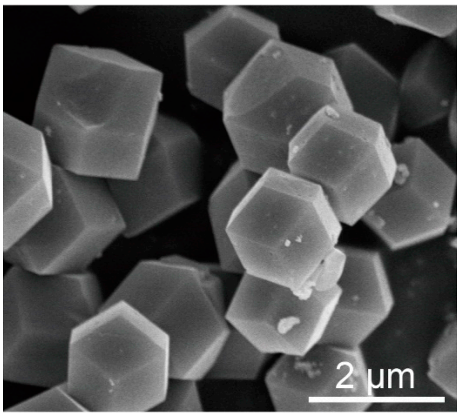

Figure S1. SEM image of Co/Ce-MOFs with Ce ratio of 0.2.

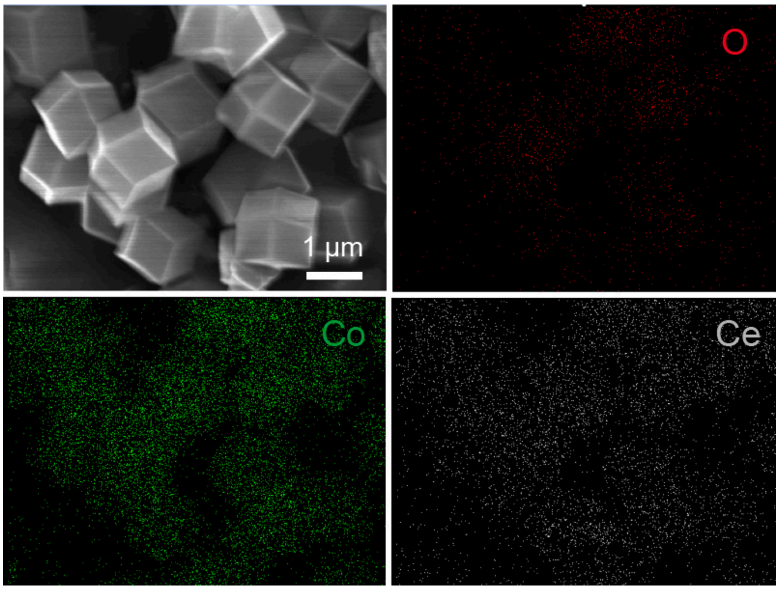

Figure S2. SEM-mapping images of Co/Ce-MOFs with Ce ratio of 0.2

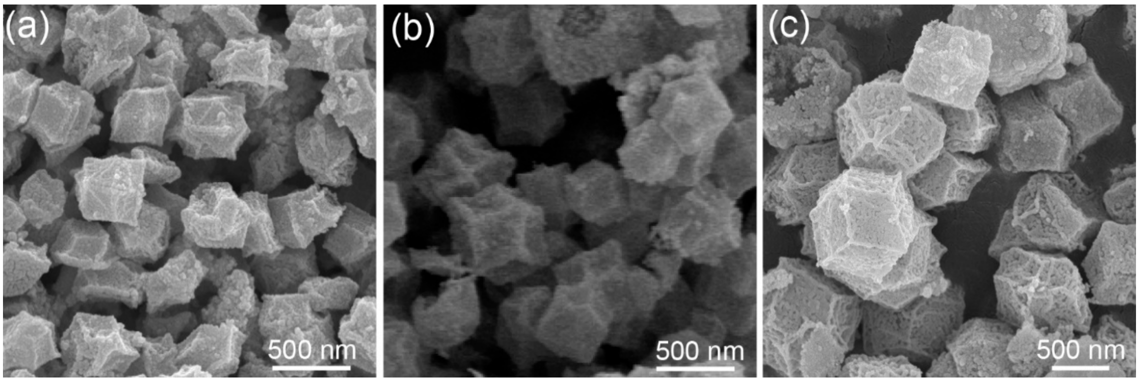

Figure S3. SEM image of (a) Co/Ce0.2-300-COHDs, (b) Co/Ce0.2COHDs and (c) Co/Ce0.2-400-COHDs.

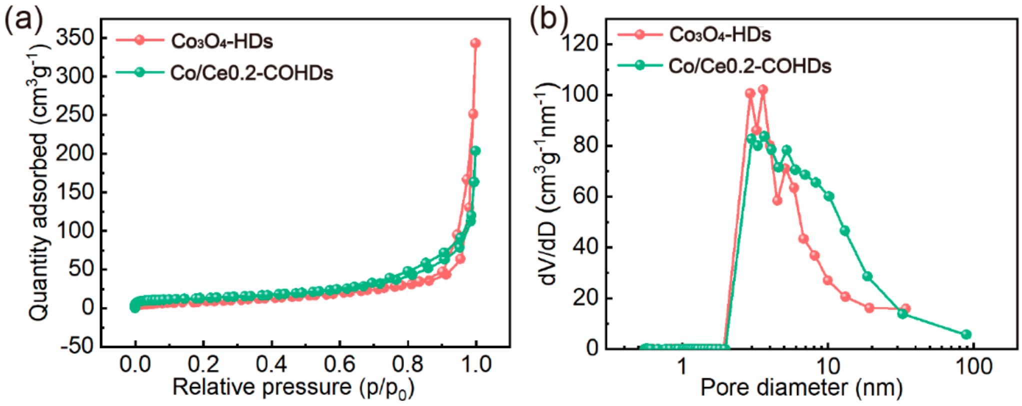

Figure S4. (a) N_2 adsorption-desorption isotherms and (b) pore size distribution curves of Co/Ce0.2-COHDs and Co_3O_4-HDs.

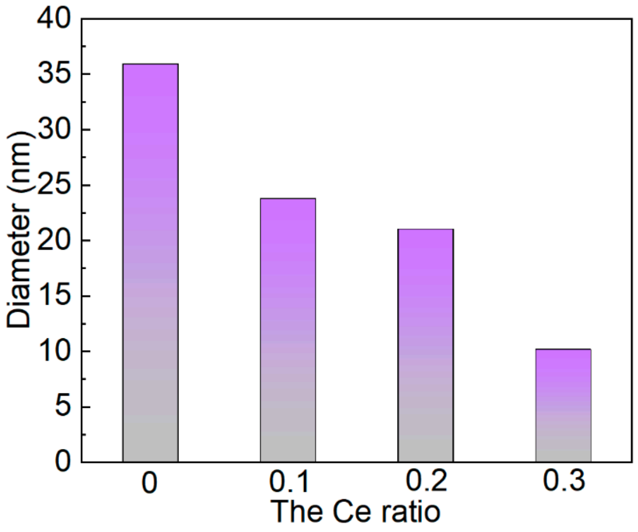

Figure S5. Grain size histogram of Co/Ce*x*-COHDs.

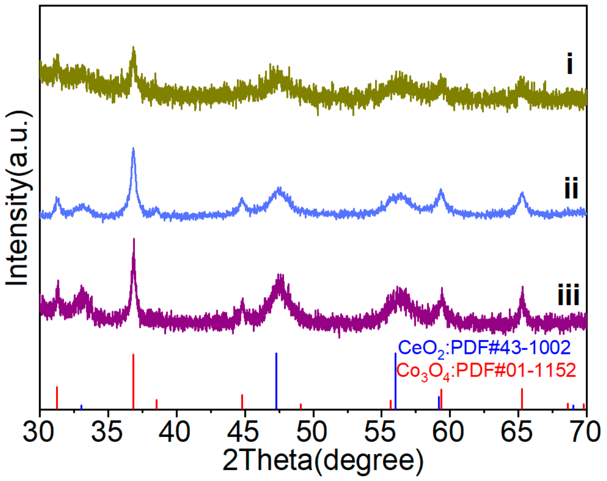

Figure S6. XRD patterns of Co/Ce0.2-300-COHDs, Co/Ce0.2COHDs, Co/Ce0.2-400-COHDs.

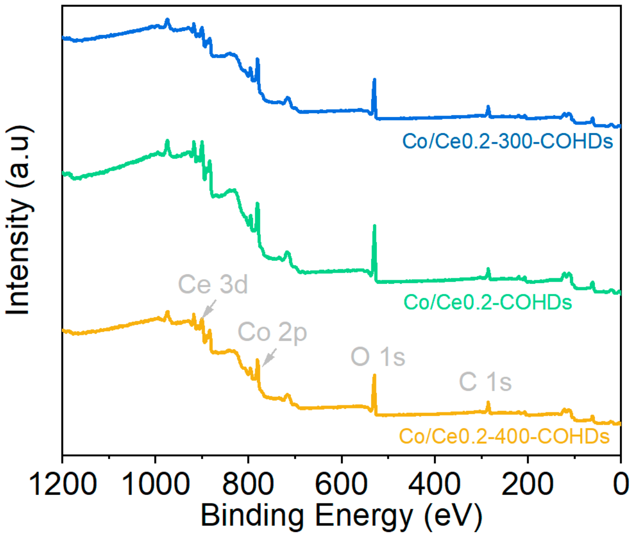

Figure S7. XPS survey spectra of Co/Ce0.2-300-COHDs, Co/Ce0.2COHDs and Co/Ce0.2-400-COHDs.

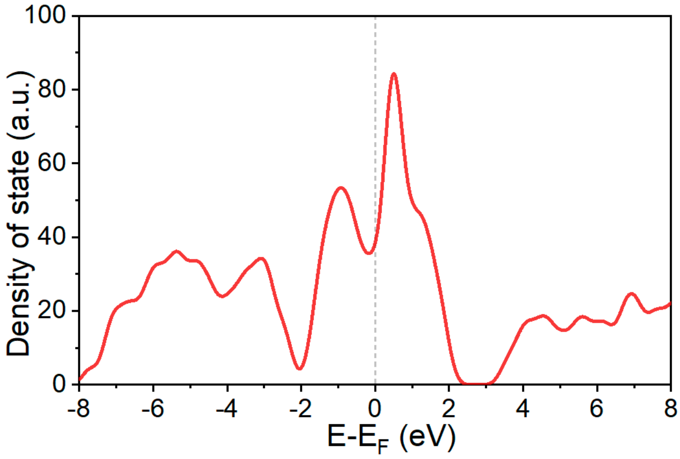

Figure S8. DOS of the Co/Ce0.2-COHDs.

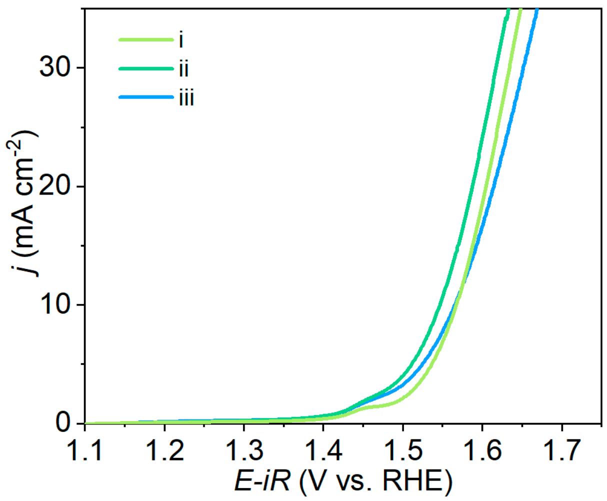

Figure S9. The polarization curves of Co/Ce0.2-COHDs with different loading (i. 0.721 mg·cm^{-2}, ii 0.961mg·cm^{-2}. iii. 1.197 mg·cm^{-2}).

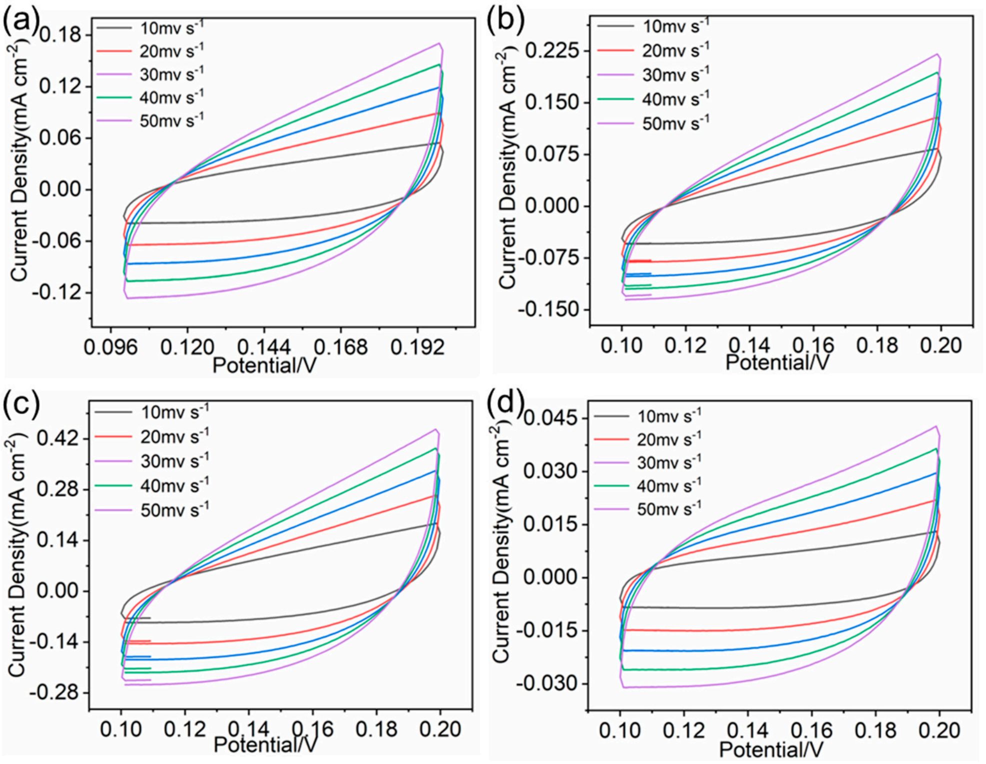

Figure S10. CVs at different scan rates of in a potential window where no Faradaic processes occur (0.1-0.2 V vs. RHE) for: (a) Co_3O_4-HDs, (b) Co/Ce0.1-COHDs, (c) Co/Ce0.2-COHDs, (d) Co/Ce0.3-COHDs.

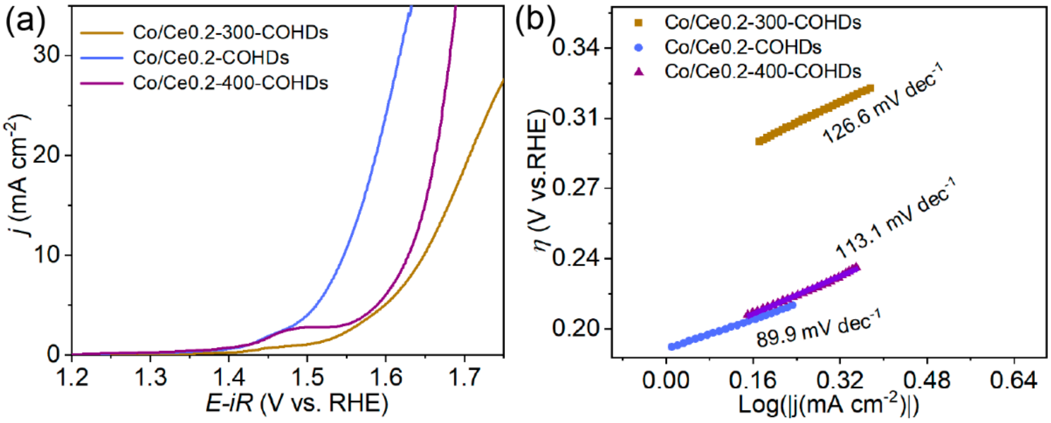

Figure S11. (a) LSV curves and (b) Tafel plots of Co/Ce0.2-300-COHDs, Co/Ce0.2COHDs and Co/Ce0.2-400-COHDs.

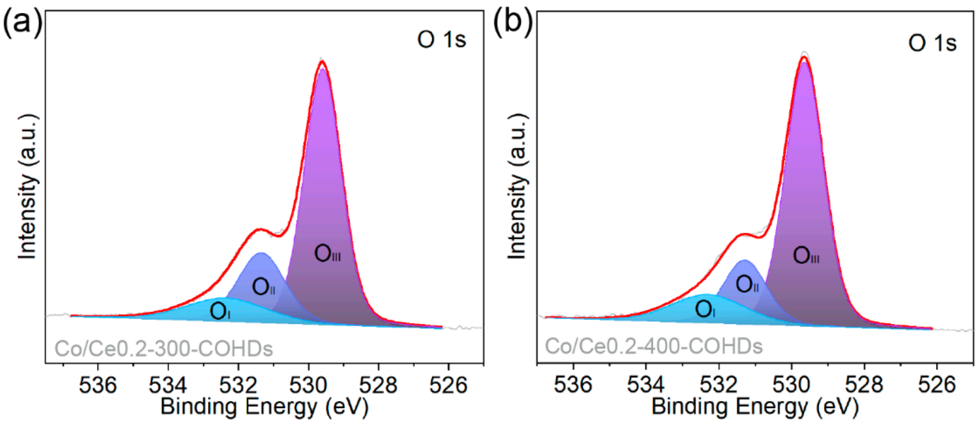

Figure S12. O 1s spectra of of (a) Co/Ce0.2-300-COHDs and (b) Co/Ce0.2-400-COHDs.

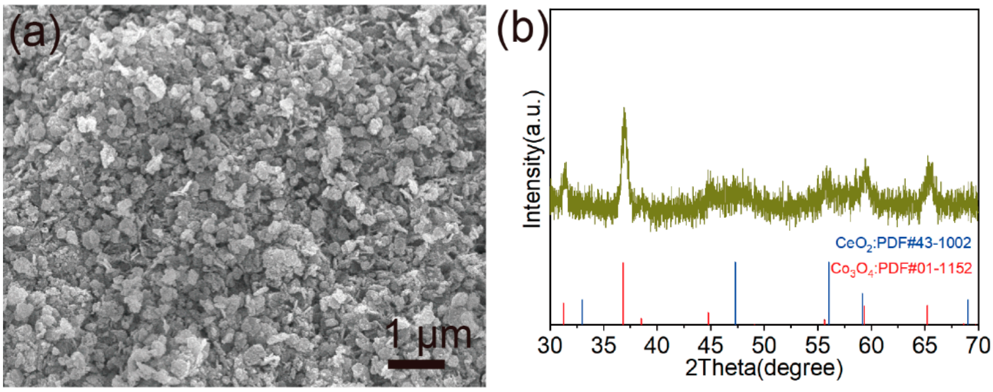

Figure S13. (a) SEM image and (b) XRD pattern of Co/Ce0.2-CONPs.

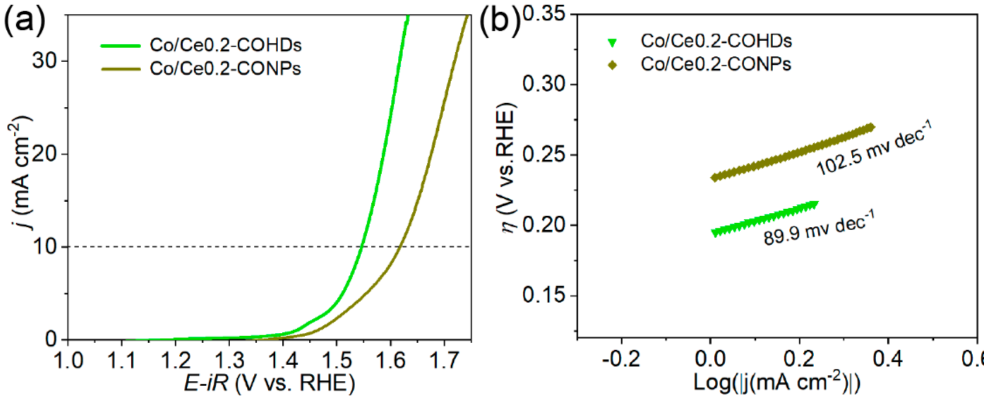

Figure S14. (a) LSV curves and (b) Tafel plots of as-prepared Co/Ce0.2-COHDs and Co/Ce0.2-CONPs.

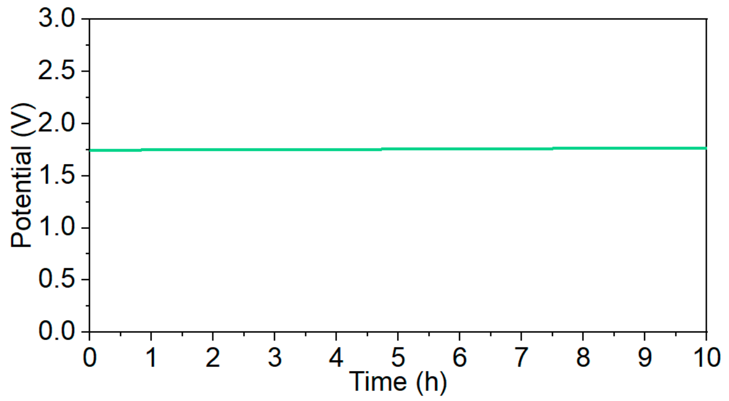

Figure S15. Long-term stability test of Co/Ce0.2COHDs.

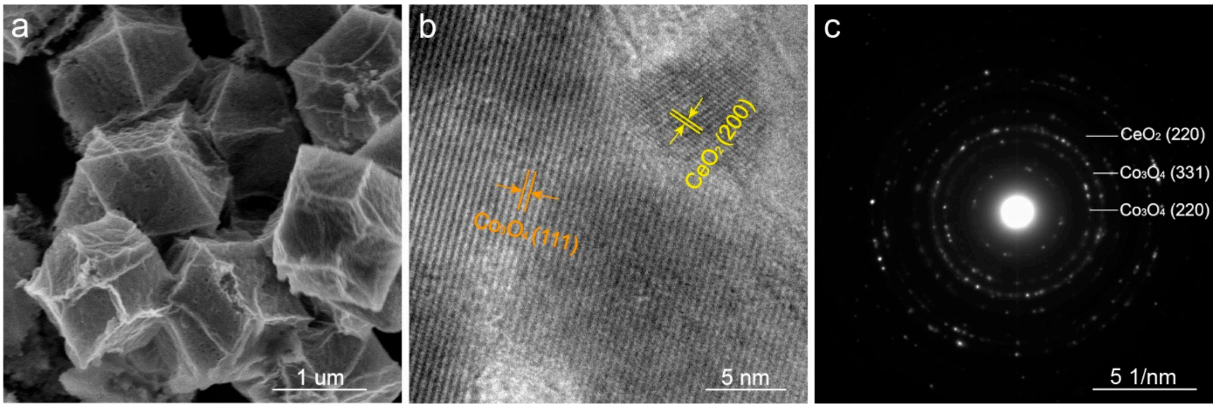

Figure S16. (a) SEM, (b) HRTEM and (c) SAED images of Co/Ce0.2COHDs after reaction.

Table S1. The TOF values of Co/Ce*x*-COHDs with different Ce ratios.

Material	TOF×10^{-2} (s^{-1})
Co_3O_4-HDs	1.75
Co/Ce0.1-COHDs	12.05
Co/Ce0.2-COHDs	16.93
Co/Ce0.3-COHDs	0.29

Table S2. Mass activity of Co/Ce*x*-COHDs with different Ce ratios at a given voltage.

Material	Mass activity(A·g^{-1})
Co_3O_4-HDs	1.89
Co/Ce0.1-COHDs	13.21
Co/Ce0.2-COHDs	18.78
Co/Ce0.3-COHDs	0.33

Table S3. O_{II}% of Co/Ce0.2-300-COHDs, Co/Ce0.2-COHDs and Co/Ce0.2-400-COHDs.

Sample	O_{II}%
Co/Ce0.2-300-COHDs	35.1
Co/Ce0.2-COHDs	32.1
Co/Ce0.2-400-COHDs	27.8

Table S4. Comparison of Tafel slope and overpotential (10 mA·cm^{-2}) with the catalysts in literature.

Materials	Electrolyte	Overpotential /mV	Tafel slope /mV·dec^{-1}	Reference
CeO_2/Ni-TMO	1.0 M KOH	350	38	[S1]
Co/CoP-5	1.0 M KOH	340	79.5	[S2]
CuO_x–CeO_2/C	0.1 M KOH	580	177	[S3]
C-Co/Co_3O_4	1.0 M KOH	352	80	[S4]
Ce–NiO–E	1.0 M KOH	382	119	[S5]
Co@NCNT	1.0 M KOH	429	116	[S6]
$La_{1-x}Ce_xCoO_3$	1.0 M KOH	380	80	[S7]
CoO_x/CeO_2/ RGO	1.0 M KOH	360	86	[S8]
Ce-$MnCo_2O_4$-3%	1.0 M KOH	390	125	[S9]
Co_3O_4/$NiCo_2O_4$	1.0 M KOH	~400	110	[S10]
Co/Ce0.2-COHDs	1.0 M KOH	316	89.9	This work

Table S5. The amount of each raw material in the precursor solution with different Ce ratios.

sample	CH_3OH /mL	$Ce(NO_3)_3\cdot 6H_2O$ /g	$Co(NO_3)_2\cdot 6H_2O$ /g	$C_4H_6N_2$ /g
Co_3O_4-HDs	120	0	0.582 g	0.656
Co/Ce0.1-COHDs	120	0.524	0.087 g	0.656
Co/Ce0.2-COHDs	120	0.466	0.174 g	0.656
Co/Ce0.3-COHDs	120	0.407	0.261 g	0.656

References

[S1] Long X.; Lin H.; Zhou D.; An Y.; Yang S. Enhancing full water-splitting performance of transition metal bifunctional electrocatalysts in alkaline solutions by tailoring CeO_2-transition metal oxides-Ni nanointerfaces, *ACS Energy Lett*. **2018**, *3*, 290–296.

[S2] Xue Z.H.; Su H.; Yu Q.Y.; Zhang B.; Wang H.H.; Li X.H.; Chen J.S. Janus Co/CoP nanoparticles as efficient mott-schottky electrocatalysts for overall water splitting in wide pH range, *Adv. Energy Mater*. **2017**, *7*, 1602355.

[S3] Goswami C.; Yamada Y.; Matus E.V.; Ismagilov I.Z.; Kerzhentsev M.; Bharali P. Elucidating the role of oxide-oxide/carbon interfaces of CuO_x-CeO_2/C in boosting Electrocatalytic Performance, *Langmuir* **2020**, *36*, 15141–15152.

[S4] Hang L.; Sun Y.; Men D.; Liu S.; Zhao Q.; Cai W.; Li Y. Hierarchical micro/nanostructured C doped Co/Co_3O_4 hollow spheres derived from PS@$Co(OH)_2$ for the oxygen evolution reaction, *J. Mater. Chem. A* **2017**, *5*, 11163-11170.

[S5] Gao W.; Xia Z.M.; Cao F.X.; Ho J.C.; Jiang Z.; Qu Y.Q. Comprehensive understanding of the spatial configurations of CeO_2 in NiO for the electrocatalytic oxygen evolution reaction: embedded or surface-loaded. *Adv. Funct. Mater.* **2018**, *28*, 1706056.

[S6] Zhang E.; Xie Y.; Ci S.; Jia J.; Cai P.; Yi L.; Wen Z. Multifunctional high-activity and robust electrocatalyst derived from metal-organic frameworks, *J. Mater. Chem. A* **2016**, *4*, 17288-17298.

[S7] Ji D.; Liu C.; Yao Y.; Luo L.; Wang W.; Chen Z. Cerium substitution in $LaCoO_3$ perovskite oxide as bifunctional electrocatalysts for hydrogen and oxygen evolution reaction, *Nanoscale* **2021**, *13*, 9952–9959.

[S8] Zhong H.; Alberto Estudillo-Wong L.; Gao Y.; Feng Y.; Alonso-Vante N. Oxygen vacancies engineering by coordinating oxygen-buffering CeO_2 with CoO nanorods as efficient bifunctional oxygen electrode electrocatalyst, *J. Energ. Chem*. **2021**, *59*, 615–625.

[S9] Huang X.; Zheng H.; Lu G.; Wang P.; Xing L.; Wang J.; Wang G. Enhanced water splitting electrocatalysis over $MnCo_2O_4$ via introduction of suitable Ce content, *ACS Sustainable Chem. Eng.* **2019**, *7*, 1169-1177.

[S10] Pan L.; Wang Q.; Li Y.; Zhang C. Amorphous cobalt-cerium binary metal oxides as high performance electrocatalyst for oxygen evolution reaction, *J. Catal*. **2020**, *284*, 14-21.
